# Image Quality and Radiation Dose of Conventional and Wide-Field High-Resolution Cone-Beam Computed Tomography for Cerebral Angiography: A Phantom Study

**DOI:** 10.3390/tomography9050134

**Published:** 2023-09-01

**Authors:** Satoru Kawauchi, Koichi Chida, Yusuke Hamada, Wataro Tsuruta

**Affiliations:** 1 Department of Radiology, Toranomon Hospital, 2-2-2 Toranomon, Minato-ku, Tokyo 105-8470, Japan; shibaken.shatle@kjd.biglobe.ne.jp (S.K.); yusuke71640@yahoo.co.jp (Y.H.); 2 Department of Radiological Technology, Tohoku University Graduate School of Medicine, 2-1 Seiryo, Aoba-ku, Sendai 980-8575, Miyagi, Japan; 3 Okinaka Memorial Institute for Medical Research, 2-2-2 Toranomon, Minato-ku, Tokyo 105-8470, Japan; 4 Department of Radiation Disaster Medicine, International Research Institute of Disaster Science, Tohoku University, 468-1 Aramaki Aza-Aoba, Aoba-ku, Sendai 980-0845, Miyagi, Japan; 5 Department of Endovascular Neurosurgery, Toranomon Hospital, 2-2-2 Toranomon, Minato-ku, Tokyo 105-8470, Japan; wataro@cf6.so-net.ne.jp

**Keywords:** cone-beam computed tomography (CT), image quality, radiation dose, cerebral angiography, disaster medicine, resilience, interventional neuroradiology (INR)

## Abstract

There has been an increase in the use of interventional neuroradiology procedures because of their non-invasiveness compared to surgeries and the improved image quality of fluoroscopy, digital subtraction angiography, and rotational angiography. Although cone-beam computed tomography (CBCT) images are inferior to multi-detector CT images in terms of low-contrast detectability and lower radiation doses, CBCT scans are frequently performed because of their accessibility. This study aimed to evaluate the image quality and radiation dose of two different high-resolution CBCTs (HR CBCT): conventional (C-HR CBCT) and wide-field HR CBCT (W-HR CBCT). The modulation transfer function (MTF), noise power spectrum (NPS), and contrast-to-noise ratio (CNR) were used to evaluate the image quality. On comparing the MTF of C-HR CBCT with a 256 × 256 matrix and that of W-HR CBCT with a 384 × 384 matrix, the MTF of W-HR CBCT with the 384 × 384 matrix was larger. A comparison of the NPS and CNR of C-HR CBCT with a 256 × 256 matrix and W-HR CBCT with a 384 × 384 matrix showed that both values were comparable. The reference air kerma values were equal for C-HR CBCT and W-HR CBCT; however, the value of the kerma area product was 1.44 times higher for W-HR CBCT compared to C-HR CBCT. The W-HR CBCT allowed for improved spatial resolution while maintaining the image noise and low-contrast detectability by changing the number of image matrices from 256 × 256 to 384 × 384. Our study revealed the image characteristics and radiation dose of W-HR CBCT. Given its advantages of low-contrast detectability and wide-area imaging with high spatial resolution, W-HR CBCT may be useful in interventional neuroradiology for acute ischemic stroke.

## 1. Introduction

There has been an increase in the use of interventional neuroradiology (INR) procedures because of their non-invasiveness compared to surgeries and the improved image quality of fluoroscopy, digital subtraction angiography, and rotational angiography [1,2,3,4]. Although cone-beam computed tomography (CBCT) images are inferior to multi-detector CT images in terms of low-contrast detectability and lower radiation doses, CBCT scans are frequently performed because of their accessibility [5,6,7,8,9,10]. During cerebral angiography, a CBCT is performed without transferring the patient to the CT room. Therefore, it is important to evaluate the radiation doses to patients in INR [11,12].

The high-resolution CBCT (HR CBCT) in cerebral angiography is useful for evaluating the intracranial stent expansion in cases of stent-assisted coil embolization and for visualization of the intracranial major/microvessels [13,14,15]. The HR CBCT with contrast media in patients with acute ischemic stroke (AIS-CBCT) is an efficient method owing to its ability to visualize the vessels distal to the occlusion site and the collateral vessels [16,17]. The conventional HR CBCT (C-HR CBCT) has a limited field of view (FOV) with a diagonal size of 22 cm; however, a 27-cm diagonal wide-area HR CBCT (W-HR CBCT) is available and expected to be clinically useful. The W-HR CBCT is a useful imaging method in AIS-CBCT, where the entire head is the object of observation, from the perspective of being able to image large areas.

When using an intracranial stent in HR-CBCT, the image reconstruction conditions require the highest possible spatial resolution of the CBCT image because it is necessary to visualize the fine stent structure [14,15]. On the contrary, the contrast dilution concentration in the vessel lumen is ultra-low for AIS-CBCT relative to HR CBCT, which uses undiluted contrast media. Therefore, the image reconstruction conditions prioritize spatial resolution resulting in noise and decreased contrast detectability. This results in a decrease in the overall image quality; therefore, it is necessary to determine the optimal image reconstruction conditions for AIS-CBCT. In INR, the number of HR CBCT scans has been increasing, and the estimation of the radiation dose to the patient is important [18,19,20,21,22,23]. An increase in the FOV size may affect the spatial resolution, noise characteristics, low-contrast detectability, and radiation dose to the patients. Although some reports have described the physical evaluation, and radiation dose of C-HR CBCT, cerebral angiography, and computed tomography [20,22,23,24,25,26,27,28], there are no reports on W-HR CBCT. The purpose of this study was two-fold: to evaluate the image quality and radiation dose of W-HR CBCT, compare it with C-HR CBCT, and determine the optimal image reconstruction for AIS-CBCT.

## 2. Materials and Methods

The study was conducted using a phantom imaging experiment; therefore, approval from an institutional review board was not required.

### 2.1. HR CBCT Protocol and Image Reconstruction

A biplane X-ray device (Azurion7 B20/15; Philips Healthcare, Best, The Netherlands) equipped with flat panel detectors was used in this study. Two types of HR CBCT protocols with different detector FOV sizes were used to evaluate the image quality. The scan protocols for HR-CBCT were as follows: tube voltage = 80 kVp; tube current = 250 mA; additional filter = none; frame rate = 30 frames/s; source-to-image distance = 120 cm; scan time = 20.8 s; and X-ray tube rotation angle = 240°. The scan conditions for C-HR CBCT and W-HR CBCT were the same, except for the detector FOV size (C-HR CBCT: 22 cm diagonal and W-HR CBCT: 27 cm diagonal).

The projection data of the HR-CBCTs were reconstructed using the following parameters: slice thickness = 5.0 mm, reconstruction kernel = stent, and image matrix number = 256 × 256, 384 × 384, and 512 × 512.

### 2.2. Spatial Resolution

A phantom (Catphan 700 Phantom; Phantom Laboratory, Salem, NY, USA) was used for the C-HR and W-HR CBCT. To evaluate the high-contrast detectability, a high-contrast module (CTP 714 module of Catphan) with fine slit-line pairs (LPs) was assessed, focusing on the identification limits of the LP module.

The spatial resolution was quantitatively assessed by measuring the modulation transfer function (MTF) using the CTP 682 module with a tungsten wire. The values of 50% and 10% were calculated from the MTF curve data.

### 2.3. Noise Characteristics

To evaluate the frequency characteristics of the image noise, we calculated the noise power spectrum (NPS) using the virtual slit method with a CTP 715 module. The NPS curve was obtained from the central (256 × 256 matrix) region of interest (ROIs) used to analyze the image noise.

### 2.4. Low-Contrast Detectability

**Figure 1:**
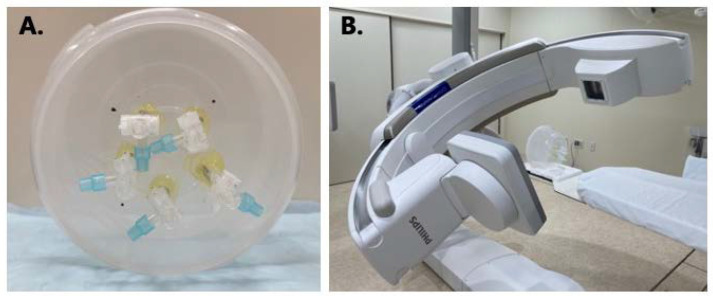
(**A**) Homemade phantom for evaluating the low-contrast detectability. (**B**) Total experimental setup of phantom measurement.

To evaluate the low-contrast detectability, the contrast-to-noise ratio (CNR) was calculated using a homemade phantom. The homemade phantom comprised cylindrical rods with a diameter of 7 mm implanted in diluted contrast media and an overall diameter of 230 mm. The rods contained 300 mgI/mL of contrast medium diluted 50, 75, 100, 125, and 150 times (Figure 1). The contrast media dilutions were selected based on the clinical image of AIS-CBCT.

**Figure 2:**
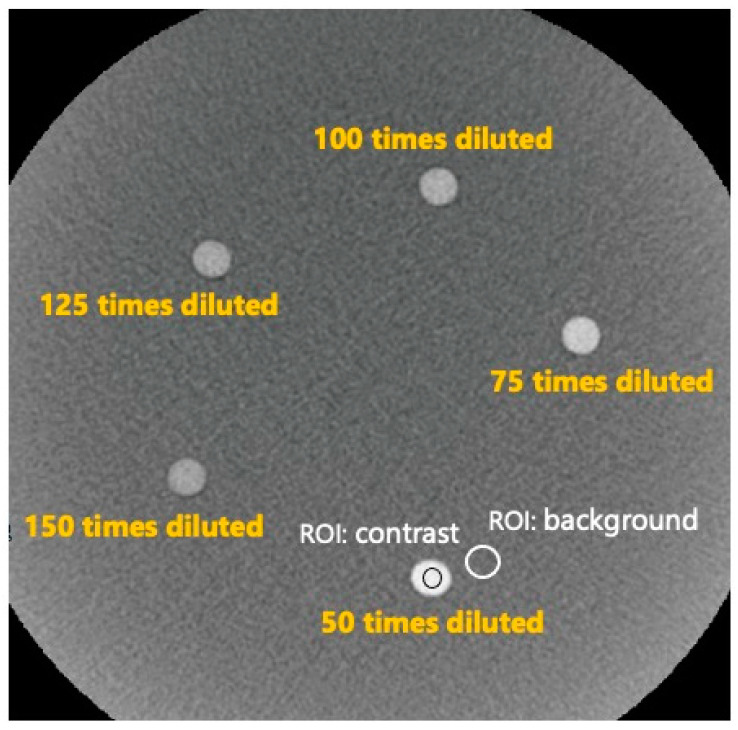
CBCT image of the homemade phantom. Each rod contained 50-, 75-, 100-, 125-, and 150-fold dilutions of the contrast media. The contrast-to-noise ratio was defined as the pixel value minus the background pixel value divided by the background standard deviation.

The CNR was calculated using the following equation:

(1) $$\mathrm{CNR}=\frac{{\mathrm{Mean}}_{\mathrm{contrast}}-{\mathrm{Mean}}_{\mathrm{BG}}}{{\mathrm{Noise}}_{\mathrm{BG}}}$$

where the Mean_contrast_ and Mean_BG_ are the pixel values measured in the rod and background ROIs, respectively (Figure 2).

### 2.5. Radiation Dose

The values of the reference air kerma (K_a,r_) and kerma area product (P_KA_) recorded by the angiographic machine were analyzed when C-HR and W-HR CBCT were performed on the Catphan phantom.

## 3. Results

### 3.1. Spatial Resolution

**Figure 3:**
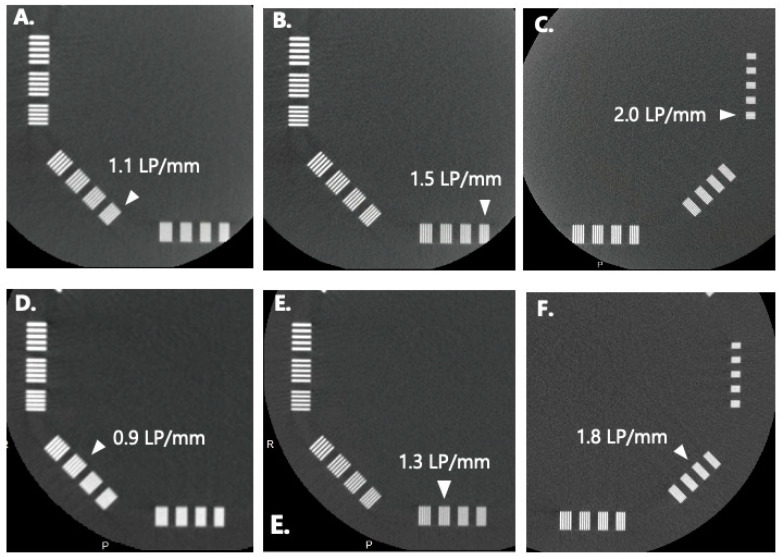
CBCT images of the high-contrast module (CTP528) of Catphan phantom. (**A**) C-HR CBCT, matrix: 256 × 256; (**B**) C-HR CBCT, matrix: 384 × 384; (**C**) C-HR CBCT, matrix: 512 × 512; (**D**) W-HR CBCT, matrix: 256 × 256; (**E**) W-HR CBCT, matrix: 384 × 384; (**F**) W-HR CBCT, matrix: 512 × 512.

Figure 3 shows the phantom images of the high-contrast module (CTP528) of the Catphan phantom. In the visual assessment of C-HR CBCT, the minimum distinguishable line pair (LP) slits at matrix numbers 256 × 256, 384 × 384, and 512 × 512 were 1.1 LP/mm, 1.5 LP/mm, and 2.0 LP/mm, respectively. In the visual assessment of W-HR CBCT, the minimum distinguishable LP slits at matrix numbers 256 × 256, 384 × 384, and 512 × 512 were 0.9 LP/mm, 1.3 LP/mm, and 1.8 LP/mm, respectively.

**Figure 4:**
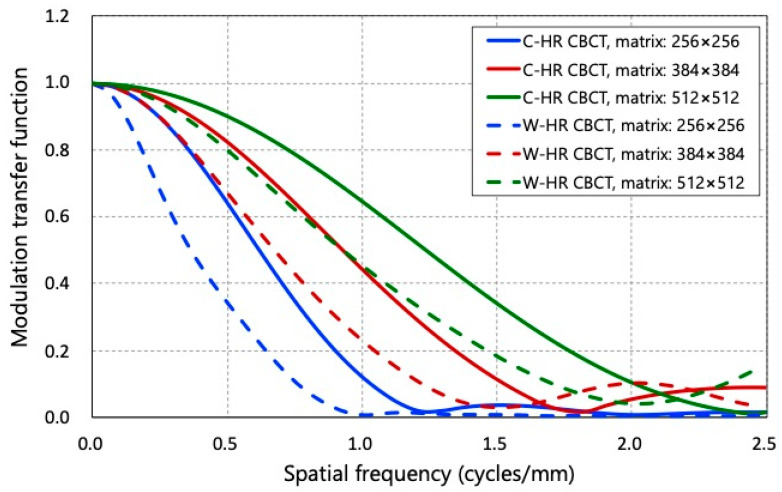
The modulation transfer function for C-HR CBCT and W-HR CBCT at each reconstruction matrix.

Figure 4 and Table 1 show the MTF curves and the 50% MTF and 10% MTF values for each matrix number in C-HR and W-HR CBCT. Compared to the MTFs for the same scan method, the MTFs improved as the number of matrices increased. The MTFs of W-HR CBCT were worse than those of C-HR CBCT when comparing the MTFs for the same number of matrices. On comparing the MTF of the C-HR CBCT with a 256 × 256 matrix and that of the W-HR CBCT with a 384 × 384 matrix, the MTF of the W-HR CBCT with a 384 × 384 matrix was improved.

### 3.2. Noise Characteristics

**Figure 5:**
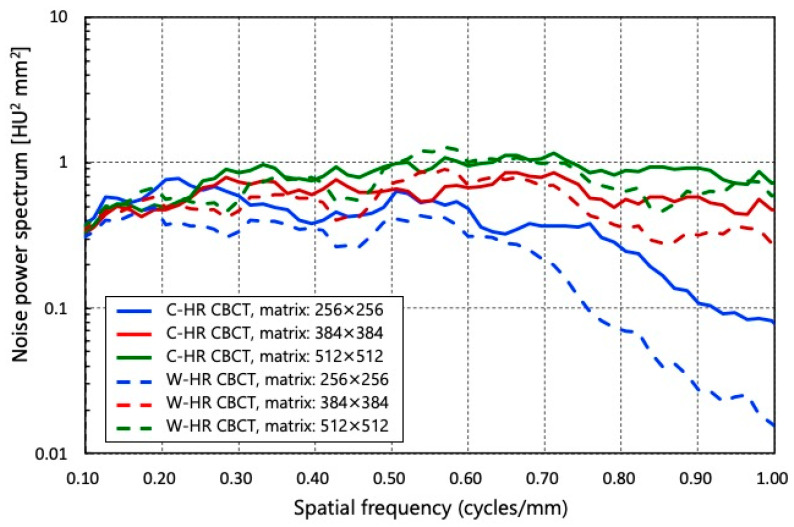
The noise power spectrum for C-HR CBCT and W-HR CBCT at each reconstruction matrix.

The NPSs of each matrix in C-HR and W-HR CBCT are shown in Figure 5. Compared to the NPS for the same scan method, the NPS increased as the number of matrices increased. When comparing the NPS for the same number of matrices, it decreased when the scan method was changed from C-HR to W-HR CBCT. On comparing the NPS of C-HR CBCT with a 256 × 256 matrix and that of W-HR CBCT with a 384 × 384 matrix, the NPS of W-HR CBCT with a 384 × 384 matrix was slightly larger in the mid-to high-frequency bands. However, both were equivalent in the low-frequency bands.

### 3.3. Low-Contrast Detectability

**Figure 6:**
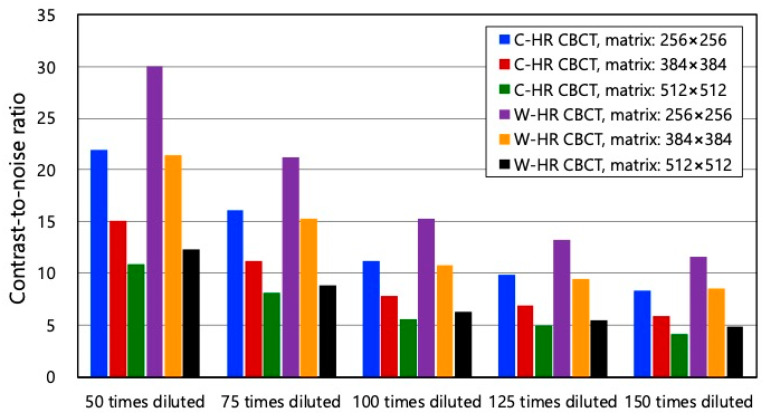
The contrast-to-noise ratio for C-HR CBCT and W-HR CBCT at each reconstruction matrix.

Figure 6 shows the CNRs of each matrix in C-HR and W-HR CBCT. The CNRs decreased as the dilution factor of the contrast medium increased. On comparing the CNRs for the same number of matrices between C-HR and W-HR CBCT, the CNR of W-HR CBCT improved. The CNRs of the C-HR CBCT with a 256 × 256 matrix and those of the W-HR CBCT with a 384 × 384 matrix at each contrast dilution concentration were comparable.

### 3.4. Radiation Dose

Table 2 shows the radiation doses of C-HR and W-HR CBCT. The K_a,r_ and P_KA_ values of the C-HR CBCT were 157 mGy and 12.8 Gy·cm^2^. The K_a,r_ and P_KA_ values of the W-HR CBCT were 157 mGy and 18.4 Gy·cm^2^. The values of K_a,r_ were equal between C-HR CBCT and W-HR CBCT; however, the P_KA_ was 1.44 times higher in W-HR CBCT than in C-HR CBCT.

## 4. Discussion

In this study, W-HR CBCT, which has a wider FOV size than C-HR CBCT, was evaluated for application in neuro-CBCT imaging in terms of technical performance (spatial resolution, noise characteristics, low-contrast detectability, and radiation dose) using a Catphan and a homemade phantom. To the best of our knowledge, this is the first study to evaluate the image quality and radiation dose of W-HR CBCT.

The spatial resolution was evaluated by a visual assessment of the high-contrast module of the Catphan phantom and the MTF. The discrimination limits of the high-contrast module and the 10% MTF values were generally in agreement. On comparing C-HR and W-HR CBCT, the spatial resolution of the W-HR CBCT decreased for the same number of matrices. This reflected the difference in the pixel binning process owing to the change in FPD size: 1 × 1 binning for C-HR CBCT and 2 × 2 binning for W-HR CBCT. The C-HR CBCT is a modification of the standard CBCT protocol used during and after cerebral angiography to assess the brain parenchyma, which provides a small FOV but a high-spatial-resolution X-ray image without pixel binning. The physicians should be aware that, while the FOV size increases with W-HR CBCT, the spatial resolution of the images also changes.

In the evaluation of the noise characteristics with low-contrast detectability, the NPS and CNR values were higher for W-HR CBCT compared to C-HR CBCT in all the reconstruction matrices. We believe that a change in the FOV size results in an increase in the matrix size and a decrease in the amount of noise contained in a pixel. When considering the use of W-HR CBCT in AIS CBCT, the reduction in image noise and improvement in low-contrast detectability are beneficial factors that contribute to improved image quality. This is because the tube current and scan time are constant regardless of the object size.

Regarding image reconstruction conditions in AIS-CBCT, Iwasaki et al. reported the usefulness of reconstructed images with a 256 × 256 matrix [17]. As AIS-CBCT images require the assessment of the lumen of major vessels and perforators, it is necessary to acquire images with the highest possible spatial resolution. As the CNR of the 256 × 256 matrix in C-HR CBCT and the 384 × 384 matrix in W-HR CBCT were equivalent, it was possible to improve the spatial resolution while maintaining a low-contrast detectability in W-HR CBCT.

Regarding the noise characteristics, the NPS of W-HR CBCT with a 384 × 384 matrix was slightly larger in the mid-to-high frequency bands; however, both were equivalent in the low-frequency band below 0.50 cycles/mm. In AIS-CBCT, it is important to assess the large vessels between 1 and 4 mm in diameter, corresponding to a spatial frequency band below 0.50 cycles/mm. Overall, the change from a 256 × 256 matrix in C-HR CBCT to a 384 × 384 matrix in W-HR CBCT resulted in improved spatial resolution without affecting noise characteristics and low-contrast detectability. The image reconstruction using 2 × 2 binning in W-HR CBCT results in a decrease in spatial resolution, which can be compensated by increasing the number of reconstruction matrices.

A radiation dose analysis was performed using the K_a,r_ and P_KA_, provided by an angiographic machine. Although the values of K_a,r_ were identical between C-HR and W-HR CBCT, the value of P_KA_ was 1.44 times higher in W-HR CBCT compared to C-HR CBCT. This is because K_a,r_ reflects the air kerma at the patient entrance reference point, and the scan conditions are the same except for the FOV size. Furthermore, P_KA_ is defined as the product of the air kerma and irradiation field area, which was larger in W-HR CBCT with a larger FOV size. Several skin injuries due to radiation have been reported in the field of INR [29,30,31,32]. These factors are associated with prolonged fluoroscopy, increased DSA exposure, and repeated procedures [33,34,35,36,37]. The International Commission on Radiological Protection Publication 118 reported that the threshold for the absorbed dose was 500 mGy [38]. This report suggested that in INR, appropriate radiation protection is required not only for the skin but also for the lens. The contribution of CBCT to the total lens dose has been estimated to be non-negligible, and the methods for lens protection during CBCT have been reported [20,22,23]. Several studies have reported that tube current modulation, organ-based tube current modulation, gantry tilt, and shielding methods with protective materials protect the lenses in head multidetector CT scans [39,40,41,42,43,44,45,46]. In neuro-CBCT, scan conditions such as the tube voltage, tube current, and additional filters are part of a fixed protocol, and the shielding method is the only method to protect the lens. As W-HR CBCT contributes more to radiation exposure compared to C-HR CBCT, appropriate radiation protection must also be considered.

In summary, in recent years, the usefulness of mechanical thrombectomy for AIS has been widely reported, and AIS-CBCT has become an essential imaging tool [16,17]. The INR procedures, including mechanical thrombectomy, tend to be complex and may sometimes increase the fluoroscopy time and resultant radiation doses to both the patients and staff [47,48,49,50,51,52,53,54,55,56,57,58,59,60]. W-HR CBCT, which has wide-area HR CBCT, is available in cerebral angiography. It is expected to be clinically useful, especially for cases of AIS. However, the image quality and the radiation dose of W-HR CBCT are unknown. Therefore, we investigated the image quality and radiation dose of W-HR CBCT and C-HR CBCT and determined the optimal image reconstruction for AIS-CBCT. The MTF, NPS, and CNR were used to evaluate the image quality. The MTF of W-HR CBCT with a 384 × 384 matrix was larger than that of C-HR CBCT with a 256 × 256 matrix. The NPSs and CNRs of C-HR CBCT with a 256 × 256 matrix and W-HR CBCT with a 384 × 384 matrix were comparable. The values of K_a,r_ were equal for C-HR CBCT and W-HR CBCT; however, the value of the P_KA_ was 1.44 times higher for W-HR CBCT than for C-HR CBCT. A wider FOV increases the area that can be imaged. This is beneficial for the assessment of collateral vessels and the vessels distal to the occlusion, which is important for AIS. In patients with AIS, a rapid procedure is required because the duration from disease onset to recanalization is related to patient outcomes [61,62]. The AIS-CBCT may shorten the duration from puncture to recanalization because it provides sufficient information to guide the selection of treatment strategies. We believe that further reductions in the duration of procedures and improved outcomes are expected with the use of W-HR CBCT for the treatment of AIS. The W-HR CBCT can be a useful imaging tool in INR procedures for AIS.

## 5. Conclusions

This study investigated the image quality and radiation dose of two different HR CBCT protocols (C-HR CBCT and W-HR CBCT) used in cerebral angiography. The W-HR CBCT allows improved spatial resolution while maintaining image noise and low-contrast detectability by changing the number of image matrices from 256 × 256 to 384 × 384. As influenced by the expansion of the FOV size, the value of P_KA_ was larger in W-HR CBCT compared to C-HR CBCT. Our study revealed the image characteristics and radiation dose of W-HR CBCT. The W-HR CBCT is useful for INR in patients with AIS, as it improves the image quality of the phantom and increases the coverage for imaging.

## Figures and Tables

**Table 1:** The value of 50% MTF and 10% MTF for C-HR CBCT and W-HR CBCT.

	Matrix	50% MTF	10% MTF
C-HR CBCT	256 × 256	0.620	1.031
	384 × 384	0.929	1.527
	512 × 512	1.240	2.015
W-HR CBCT	256 × 256	0.366	0.760
	384 × 384	0.675	1.218
	512 × 512	0.929	1.705

**Table 2:** Radiation doses for C-HR CBCT and W-HR CBCT.

	Reference Air-Kerma (mGy)	Kerma Area Product (Gy·cm^2^)
C-HR CBCT	157	12.8
W-HR CBCT	157	18.4

## Data Availability

The data presented in this study are available on request from the corresponding author.

## References

[1] Baek J.H., Kim B.M., Heo J.H., Kim D.J., Nam H.S., Kim Y.D., Choi H.S., Kim J.H., Kim J.W. (2020). Association between flat-panel computed tomography hyperattenuation and clinical outcome after successful recanalization by endovascular treatment. J. Neurosurg..

[2] Becske T., Kallmes D.F., Saatci I., McDougall C.G., Szikora I., Lanzino G., Moran C.J., Woo H.H., Lopes D.K., Berez A.L. (2013). Pipeline for uncoilable or failed aneurysms: Results from a multicenter clinical trial. Radiology.

[3] Chalouhi N., McMahon J.F., Moukarzel L.A., Starke R.M., Jabbour P., Dumont A.S., Tjoumakaris S., Gingold E.L., Rosenwasser R., Gonzalez L.F. (2014). Flow diversion versus traditional aneurysm embolization strategies: Analysis of fluoroscopy and procedure times. J. Neurointerv. Surg..

[4] Sciacca S., Bassiouny A., Mansoor N., Minett T., Balasundaram P., Siddiqui J., Joshi Y., Derakhshani S., Kandasamy N., Booth T.C. (2023). Early Outcomes of the Pipeline Vantage Flow Diverter: A Multicentre Study. Clin. Neuroradiol..

[5] Bai M., Liu B., Mu H., Liu X., Jiang Y. (2012). The comparison of radiation dose between C-arm flat-detector CT (DynaCT) and multi-slice CT (MSCT): A phantom study. Eur. J. Radiol..

[6] Cancelliere N.M., Hummel E., van Nijnatten F., van de Haar P., Withagen P., van Vlimmeren M., Hallacoglu B., Agid R., Nicholson P., Mendes Pereira V. (2023). The butterfly effect: Improving brain cone-beam CT image artifacts for stroke assessment using a novel dual-axis trajectory. J. Neurointerv. Surg..

[7] Hosoo H., Ito Y., Marushima A., Hayakawa M., Masumoto T., Ishikawa E., Matsumaru Y. (2023). Image quality improvements for brain soft tissue in neuro-endovascular treatments: A novel dual-axis “butterfly” trajectory for optimized Cone-Beam CT. Eur. J. Radiol..

[8] Irie K., Murayama Y., Saguchi T., Ishibashi T., Ebara M., Takao H., Abe T. (2008). Dynact soft-tissue visualization using an angiographic C-arm system: Initial clinical experience in the operating room. Neurosurgery.

[9] Jones A.K., Odisio B.C. (2020). Comparison of radiation dose and image quality between flat panel computed tomography and multidetector computed tomography in a hybrid CT-angiography suite. J. Appl. Clin. Med. Phys..

[10] Sánchez R., Vañó E., Fernández J., Rosati S., López-Ibor L. (2016). Radiation doses in patient eye lenses during interventional neuroradiology procedures. Am. J. Neuroradiol..

[11] Chida K., Saito H., Otani H., Kohzuki M., Takahashi S., Yamada S., Shirato K., Zuguchi M. (2006). Relationship between fluoroscopic time, dose-area product, body weight, and maximum radiation skin dose in cardiac interventional procedures. AJR Am. J. Roentgenol..

[12] Moritake T., Matsumaru Y., Takigawa T., Nishizawa K., Matsumura A., Tsuboi K. (2008). Dose measurement on both patients and operators during neurointerventional procedures using photoluminescence glass dosimeters. AJNR Am. J. Neuroradiol..

[13] Kizilkilic O., Kocer N., Metaxas G.E., Babic D., Homan R., Islak C. (2012). Utility of VasoCT in the treatment of intracranial aneurysm with flow-diverter stents. J. Neurosurg..

[14] Raz E., Nossek E., Sahlein D.H., Sharashidze V., Narayan V., Ali A., Esparza R., Peschillo S., Chung C., Diana F. (2023). Principles, techniques and applications of high resolution cone beam CT angiography in the neuroangio suite. J. Neurointerv. Surg..

[15] Snoeren R.M., Söderman M., Kroon J.N., Roijers R.B., de With P.H., Babic D. (2012). High-resolution 3D X-ray imaging of intracranial nitinol stents. Neuroradiology.

[16] Amano T., Sato M., Matsumaru Y., Sakuma H., Yoda S., Hamada Y. (2017). Intra-arterial Contrasted Cone-beam Computed Tomography Assessment of Vessels Distal from Occluded Site in Acute Ischemic Stroke with Major Vessel Occlusion. Neurol. Med. Chir..

[17] Iwasaki M., Saito M., Nemoto A., Suzuki T., Hikita C., Fukuta S., Sato H., Morimoto M. (2019). Diluted Contrast-enhanced Cone-beam CT during Acute-phase Recanalization Therapy for Occlusion of the Middle Cerebral Artery. J. Neuroendovasc. Ther..

[18] Abuzaid M.M., Elshami W., Jayachandran D., Korappil N., Tekin H.O. (2022). Establishment of Diagnostic Reference Levels in Cone Beam Computed Tomography Scans in the United Arab Emirates. Tomography.

[19] Brendlin A.S., Estler A., Plajer D., Lutz A., Grözinger G., Bongers M.N., Tsiflikas I., Afat S., Artzner C.P. (2022). AI Denoising Significantly Enhances Image Quality and Diagnostic Confidence in Interventional Cone-Beam Computed Tomography. Tomography.

[20] Kawauchi S., Chida K., Hamada Y., Tsuruta W. (2022). Lens dose reduction with a bismuth shield in neuro cone-beam computed tomography: An investigation on optimum shield device placement conditions. Radiol. Phys. Technol..

[21] Kawauchi S., Chida K., Moritake T., Hamada Y., Matsumaru Y., Tsuruta W., Sato M., Hosoo H., Sun L. (2020). Treatment of Internal Carotid Aneurysms Using Pipeline Embolization Devices: Measuring the Radiation Dose of the Patient and Determining the Factors Affecting It. Radiat. Prot. Dosim..

[22] Kawauchi S., Chida K., Moritake T., Hamada Y., Tsuruta W. (2021). Radioprotection of eye lens using protective material in neuro cone-beam computed tomography: Estimation of dose reduction rate and image quality. Phys. Med..

[23] Kawauchi S., Chida K., Moritake T., Matsumaru Y., Hamada Y., Sakuma H., Yoda S., Sun L., Sato M., Tsuruta W. (2019). Estimation of patient lens dose associated with C-arm cone-beam computed tomography usage during interventional neuroradiology. Radiat. Prot. Dosim..

[24] Avilés Lucas P., Dance D.R., Castellano I.A., Vañó E. (2005). Estimation of the peak entrance surface air kerma for patients undergoing computed tomography-guided procedures. Radiat. Prot. Dosim..

[25] Kirisattayakul W., Pattum P., Munkong W., Prabsattroo T., Khottapat C., Chomkhunthod T., Pungkun V. (2023). Comparing Radiation Dose of Cerebral Angiography Using Conventional and High kV Techniques: A Retrospective Study on Intracranial Aneurysm Patients and a Phantom Study. Tomography.

[26] Sarti M., Brehmer W.P., Gay S.B. (2012). Low-dose techniques in CT-guided interventions. Radiographics.

[27] Tsuda N., Mitsui K., Oda S. (2016). Evaluation for Basic Image Qualities Dependence on the Position in XYZ Directions and Acquisition Parameters of the Cone Beam CT for Angiography System with Flat Panel Detector. Nihon Hoshasen Gijutsu Gakkai Zasshi.

[28] Ucar F.A., Frenzel M., Abello Mercado M.A., Altmann S., Reder S., Brockmann C., Brockmann M.A., Othman A.E. (2021). Feasibility of Ultra-High Resolution Supra-Aortic CT Angiography: An Assessment of Diagnostic Image Quality and Radiation Dose. Tomography.

[29] Herrero-Moyano M., Hernández P.M., Castañon P.G., Monreal J.L.C. (2022). Transient Rectangular Alopecia after Endovascular Embolization: A Case Series of four Patients Describing Dermoscopic and Histopathologic Findings. Int. J. Trichol..

[30] Imanishi Y., Fukui A., Niimi H., Itoh D., Nozaki K., Nakaji S., Ishizuka K., Tabata H., Furuya Y., Uzura M. (2005). Radiation-induced temporary hair loss as a radiation damage only occurring in patients who had the combination of MDCT and DSA. Eur. Radiol..

[31] Román-Sainz J., Silvestre-Torner N., Gruber-Velasco F., Romero-Jiménez B., Lobato-Berezo A., Imbernón-Moya A. (2022). Square-shaped Alopecia After Embolization of Intracranial Aneurysm: A Case Report and Review. Dermatol. Pract. Concept..

[32] Thorat J.D., Hwang P.Y. (2007). Peculiar geometric alopecia and trigeminal nerve dysfunction in a patient after Guglielmi detachable coil embolization of a ruptured aneurysm. J. Stroke Cerebrovasc. Dis..

[33] Chida K., Kaga Y., Haga Y., Kataoka N., Kumasaka E., Meguro T., Zuguchi M. (2013). Occupational dose in interventional radiology procedures. AJR Am. J. Roentgenol..

[34] Haga Y., Chida K., Kaga Y., Sota M., Meguro T., Zuguchi M. (2017). Occupational eye dose in interventional cardiology procedures. Sci. Rep..

[35] Inaba Y., Hitachi S., Watanuki M., Chida K. (2022). Radiation Eye Dose for Physicians in CT Fluoroscopy-Guided Biopsy. Tomography.

[36] Kato M., Chida K., Ishida T., Toyoshima H., Yoshida Y., Yoshioka S., Moroi J., Kinoshita T. (2019). Occupational Radiation Exposure of the Eye in Neurovascular Interventional Physician. Radiat. Prot. Dosim..

[37] Nawfel R.D., Judy P.F., Silverman S.G., Hooton S., Tuncali K., Adams D.F. (2000). Patient and personnel exposure during CT fluoroscopy-guided interventional procedures. Radiology.

[38] Stewart F.A., Akleyev A.V., Hauer-Jensen M., Hendry J.H., Kleiman N.J., Macvittie T.J., Aleman B.M., Edgar A.B., Mabuchi K., Muirhead C.R. (2012). ICRP publication 118: ICRP statement on tissue reactions and early and late effects of radiation in normal tissues and organs-threshold doses for tissue reactions in a radiation protection context. Ann. ICRP.

[39] Ciarmatori A., Nocetti L., Mistretta G., Zambelli G., Costi T. (2016). Reducing absorbed dose to eye lenses in head CT examinations: The effect of bismuth shielding. Australas. Phys. Eng. Sci. Med..

[40] Hopper K.D., Neuman J.D., King S.H., Kunselman A.R. (2001). Radioprotection to the eye during CT scanning. AJNR Am. J. Neuroradiol..

[41] Inoue Y., Itoh H., Miyatake H., Hata H., Sasa R., Shiibashi N., Mitsui K. (2022). Automatic Exposure Control Attains Radiation Dose Modulation Matched with the Head Size in Pediatric Brain CT. Tomography.

[42] Kim J.S., Kwon S.M., Kim J.M., Yoon S.W. (2017). New organ-based tube current modulation method to reduce the radiation dose during computed tomography of the head: Evaluation of image quality and radiation dose to the eyes in the phantom study. Radiol. Med..

[43] Mehnati P., Malekzadeh R., Sooteh M.Y. (2019). Use of bismuth shield for protection of superficial radiosensitive organs in patients undergoing computed tomography: A literature review and meta-analysis. Radiol. Phys. Technol..

[44] Nikupaavo U., Kaasalainen T., Reijonen V., Ahonen S.M., Kortesniemi M. (2015). Lens dose in routine head CT: Comparison of different optimization methods with anthropomorphic phantoms. AJR Am. J. Roentgenol..

[45] Ota J., Yokota H., Kobayashi T., Ogata Y., Kubo T., Chida K., Masuda Y., Uno T. (2022). Head CT dose reduction with organ-based tube current modulation. Med. Phys..

[46] Summerlin D., Willis J., Boggs R., Johnson L.M., Porter K.K. (2022). Radiation Dose Reduction Opportunities in Vascular Imaging. Tomography.

[47] Valentin J. (2007). The 2007 Recommendations of the International Commission on Radiological Protection.

[48] International Atomic Energy Agency (2014). Implications for Occupational Radiation Protection of the New Dose Limit for the Lens of the Eye.

[49] International Atomic Energy Agency (2014). Radiation Protection and Safety of Radiation Sources: International Basic Safety Standards.

[50] Chida K. (2022). What are useful methods to reduce occupational radiation exposure among radiological medical workers, especially for interventional radiology personnel?. Radiol. Phys. Technol..

[51] Endo M., Haga Y., Sota M., Tanaka A., Otomo K., Murabayashi Y., Abe M., Kaga Y., Inaba Y., Suzuki M. (2021). Evaluation of novel X-ray protective eyewear in reducing the eye dose to interventional radiology physicians. J. Radiat. Res..

[52] Fujibuchi T. (2021). Radiation protection education using virtual reality for the visualisation of scattered distributions during radiological examinations. J. Radiol. Prot..

[53] Inaba Y., Hitachi S., Watanuki M., Chida K. (2021). Occupational Radiation Dose to Eye Lenses in CT-Guided Interventions Using MDCT-Fluoroscopy. Diagnostics.

[54] Koenig A.M., Etzel R., Greger W., Viniol S., Fiebich M., Thomas R.P., Mahnken A.H. (2020). Protective Efficacy of Different Ocular Radiation Protection Devices: A Phantom Study. Cardiovasc. Intervent. Radiol..

[55] Martin C.J. (2011). A 20 mSv dose limit for the eye: Sense or no sense?. J. Radiol. Prot..

[56] Matsubara K. (2021). Assessment of Radiation Dose in Medical Imaging and Interventional Radiology Procedures for Patient and Staff Safety. Diagnostics.

[57] Matsubara K., Lertsuwunseri V., Srimahachota S., Krisanachinda A., Tulvatana W., Khambhiphant B., Sudchai W., Rehani M. (2017). Eye lens dosimetry and the study on radiation cataract in interventional cardiologists. Phys. Med..

[58] Matsunaga Y., Kawaguchi A., Kobayashi K., Kobayashi M., Asada Y., Minami K., Suzuki S., Chida K. (2016). Effective radiation doses of CT examinations in Japan: A nationwide questionnaire-based study. Br. J. Radiol..

[59] Valentin J. (2000). Avoidance of radiation injuries from medical interventional procedures, ICRP Publication 85. Ann. ICRP.

[60] Vañó E., González L., Beneytez F., Moreno F. (1998). Lens injuries induced by occupational exposure in non-optimized interventional radiology laboratories. Br. J. Radiol..

[61] Khatri P., Abruzzo T., Yeatts S.D., Nichols C., Broderick J.P., Tomsick T.A. (2009). Good clinical outcome after ischemic stroke with successful revascularization is time-dependent. Neurology.

[62] Khatri P., Yeatts S.D., Mazighi M., Broderick J.P., Liebeskind D.S., Demchuk A.M., Amarenco P., Carrozzella J., Spilker J., Foster L.D. (2014). Time to angiographic reperfusion and clinical outcome after acute ischaemic stroke: An analysis of data from the Interventional Management of Stroke (IMS III) phase 3 trial. Lancet Neurol..

